# Calcified Cartilage-Guided Identification of Osteogenic Molecules and Geometries

**DOI:** 10.1021/acsbiomaterials.3c01799

**Published:** 2024-04-18

**Authors:** Katsuhiro Kawaai, Yukiko Kuroda, Koichi Matsuo

**Affiliations:** Laboratory of Cell and Tissue Biology, Keio University School of Medicine, 35 Shinanomachi, Shinjuku, Tokyo 160-8582, Japan

**Keywords:** calcified cartilage, mineralization, surface coating, microarchitecture, micropattern

## Abstract

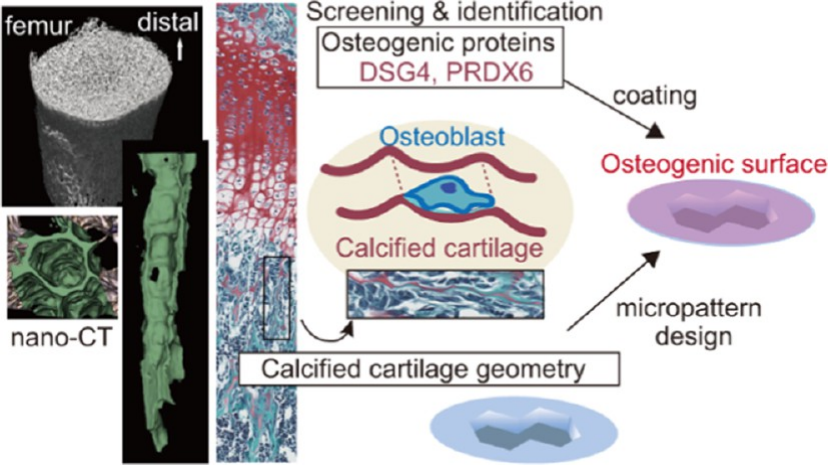

Calcified cartilage digested by chondroclasts provides an excellent scaffold to initiate bone formation. We analyzed bioactive proteins and microarchitecture of calcified cartilage either separately or in combination and evaluated biomimetic osteogenic culture conditions of surface-coated micropatterning. To do so, we prepared a crude extract from porcine femoral growth plates, which enhanced *in vitro* mineralization when coated on flat-bottom culture dishes, and identified four candidate proteins by fractionation and mass spectrometry. Murine homologues of two candidates, desmoglein 4 (DSG4) and peroxiredoxin 6 (PRDX6), significantly promoted osteogenic activity based on *in vitro* mineralization and osteoblast differentiation. Moreover, we observed DSG4 and PRDX6 protein expression in mouse femur. In addition, we designed circular, triangular, and honeycomb micropatterns with 30 or 50 μm units, either isolated or connected, to mimic hypertrophic chondrocyte-sized compartments. Isolated, larger honeycomb patterns particularly enhanced osteogenesis *in vitro*. Mineralization on micropatterns was positively correlated with the reduction of osteoblast migration distance in live cell imaging. Finally, we evaluated possible combinatorial effects of coat proteins and micropatterns and observed an additive effect of DSG4 or PRDX6 coating with micropatterns. These data suggest that combining a bioactive surface coating with osteogenic micropatterns may recapitulate initiation of bone formation during endochondral ossification.

## Introduction

*In vitro* analysis of osteoblast differentiation and osteogenesis is widely used to investigate osteoblast biology and in applied research aimed at improving bone tissue engineering. Both types of research often require culture of various osteoblastic cell lines, primary calvarial osteoblasts, or bone marrow-derived mesenchymal stem cells (MSCs) in an osteogenic medium typically containing ascorbic acid to enhance collagen synthesis and β-glycerophosphate as a phosphate source. Osteoblast differentiation is usually monitored by alkaline phosphatase (ALP) activity and expression of marker genes such as *Col1a1*, which encodes type I collagen, and *Bglap*, which encodes osteocalcin. *In vitro*, osteogenesis is typically visualized by mineralized nodule formation after alizarin red S staining.

There are two distinct types of *in vitro* analysis of osteogenesis. Biochemical analyses take a candidate gene/protein approach: namely, candidate genes are overexpressed, knocked down, or knocked out in cells, and effects on osteogenesis are quantified. Such analysis has identified stimulatory or inhibitory effects of diverse molecules assignable to bone morphogenetic protein (BMP), Wnt, or other signaling pathways. Biochemically, hypoxia also induces responses activating not only positive (HIF1) but also negative (HIF2) regulators in osteoblasts both *in vitro* and *in vivo.*(1) Also studied is oxidizing stress, which inhibits mineralization and osteogenesis.^2^ Bioactive molecules may also be provided in culture medium or coated on culture plates.

By contrast, biophysical culture approaches involve use of microprinted or nonflat surfaces that affect cell shape or surfaces with various stiffness values. For example, human MSCs complated onto large fibronectin islands but to an adipocyte fate when plated onto small fibronectin islands under an osteogenic–adipogenic mixed culture medium demonstrating the effect of geometry on osteoblast differentiation.^3^ Changes in surface curvature can be sensed by osteoblasts and guide spatiotemporal cell and tissue organization.^4,5^ Information obtained from these analyses is used to design materials for implants to facilitate bone regeneration or to improve implant osteointegration.^6^ Indeed, understanding of how surface modifications impact device efficacy may promote better treatment of bone defects using titanium implants in orthopedics and dentistry.^7^

Here, we focused on the biochemical and biophysical properties of calcified cartilage, which serves as a scaffold to initiate endochondral ossification, and how those properties may modulate bone formation by osteoblasts. In order to develop osteogenic surface mimicking calcified cartilage, we first searched osteogenic proteins and identified DSG4 and PRDX6 as candidates for osteogenic coating. We then established the assay for osteogenic geometries by using poly(dimethylsiloxane) (PDMS) micropatterns, inspired from calcified cartilage and found the structural elements to facilitate mineralization by osteoblasts. Finally, combination of osteogenic proteins and geometry mimicking of calcified cartilage achieved enhanced bone formation *in vitro*.

## Materials and Methods

### Animals

Generation of *Col1a1*-AcGFP transgenic mice, which express AcGFP driven by the *Col1a1* promoter (2.3 kbp), was previously described.^8^*TRAP*-tdTomato mice were a kind gift of Dr. Masaru Ishii (Osaka University).^9^ C57BL/6J mice were purchased from Clea-Japan. All mice were maintained under specific pathogen-free conditions, and experiments were performed in accordance with the Institutional Guidelines on Animal Experimentation at Keio University. Every effort was made to minimize the number of animals used.

### Cell Culture

Mouse preosteoblast MC3T3-E1 cells were purchased from ATCC (Subclone 4, CRL-2593) and cultured in ascorbic acid-free MEMα (A1049001, Thermo Fisher) supplemented with 10% fetal bovine serum (FBS) and a 1% penicillin–streptomycin mixture (26253-84, Nacalai Tesque). Either medium change or passage was performed every 3–4 days. GFP-MC3T3-E1 cells were established by transforming MC3T3-E1 cells with pcDNA3-EGFP using Lipofectamine LTX (A12621, Thermo Fisher) and subsequent selection of high expressors by flow cytometry. Primary calvarial osteoblasts were isolated from C57BL/6J mice at postnatal day 1 (P1) and cultured as previously reported.^10^

### Osteoblast Differentiation and Mineralization Assay

MC3T3-E1 preosteoblasts or primary mouse calvarial osteoblasts were seeded at a density of 6.5 × 10^4^ cells/cm^2^ and differentiated in the presence of 50 μg/mL magnesium l-ascorbate and 10 mM disodium β-glycerophosphate (osteoblast differentiation medium). Medium was changed every 3–4 days. After culturing 4 days in that medium, alkaline phosphatase (ALP) activity was stained using an ALP staining kit (85L1–1KT, Sigma-Aldrich). After 13–30 days of culture, mineralization nodules were stained by 0.5% Alizarin red S (A5533, Sigma-Aldrich). Photographs were obtained using Thunder Imaging Systems (Leica) and quantified with ImageJ (NIH).

### Extraction, Screening, and Identification of Osteogenic Factors from Pig Femur Growth Plate (GP)

**Figure 1:**
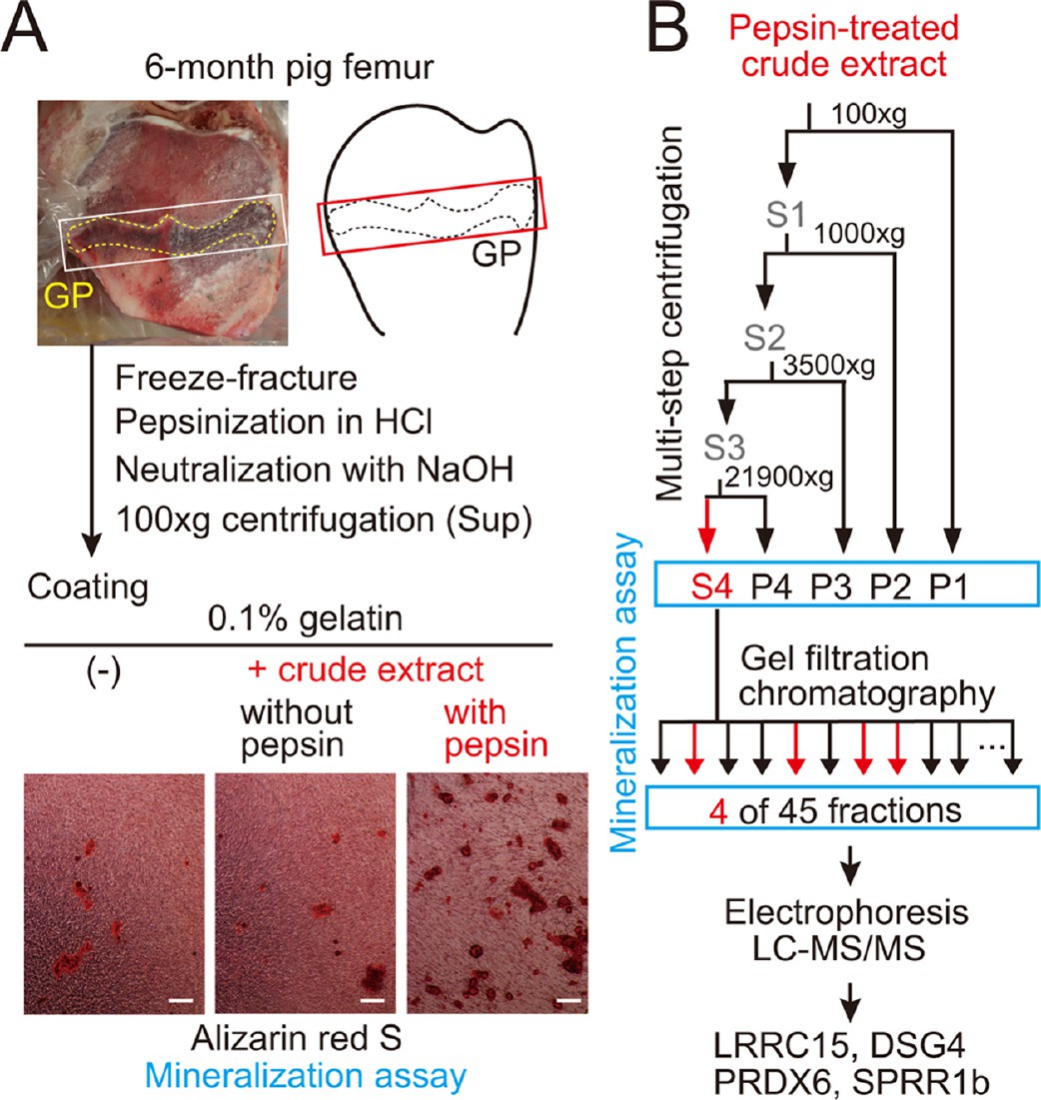
Screening for osteogenic factors from calcified cartilage of porcine femur. (A) Top: schematic showing preparation of crude extract from a femoral growth plate (GP, dotted lines) and surrounding tissue (rectangles) from 6-month-old pig. Bottom: Alizarin red S staining following a mineralization assay using the osteoblast MC3T3-E1 line. Scale, 100 μm. (B) Schematic illustrating identification of osteogenic components in supernatants (S1–S4) from the crude extract defined in (A). After resuspension and sonication in PBS, pellets (P1–P4) were also subjected to a mineralization assay. Red coloring indicates osteogenic fractions.

The epiphysis of frozen femur from a 6-month pig (TOKYO SHIBAURA ZOUKI Co., Ltd.) was cut into 2 cm squares with a saw and crushed using a cool mill (Tokken). The resulting crushed powder was mixed with 30 mg/mL pepsin in 0.01 N HCl, rotated at room temperature (RT) 48 h, and then centrifuged at 1000*g* for 20 min. The supernatant was neutralized with 10× phosphate-buffered saline (PBS) and NaOH and then aliquoted and stored as a crude extract at −80 °C for later use. Subsequently, the crude extract was separated into six fractions (S1P, S2P, S3P, S4, P1S, and P1P in Figure 1B) by multistep centrifugation (100*g*, 1000*g*, 3500*g*, and 21 900*g*) and assessed in a mineralization assay. An osteogenic fraction (S4) was subfractionated into a total of 185 samples by gel filtration using AKTA prime (Cytiva) with a Hiprep 16/60 column (Thermo Fisher; running buffer, 50 mM phosphate/150 mM NaCl). Fractions were concentrated by SpeedVac (Thermo Fisher). The 45 selected fractions were subjected to a mineralization assay, and 4 osteogenic fractions were pooled, concentrated by SpeedVac, and separated by electrophoresis. After silver staining (Silver stain MS Kit, Fujifilm Wako), bands were analyzed by mass spectrometry and proteins were identified (performed by Naoya Hatano, Okayama University).

### Expression Vectors and Protein Purification

Full-length Lrrc15 (GeneBank accession no. NM_028973.2) was amplified by polymerase chain reaction (PCR) from mouse femur cDNA using primers shown in Table S1 and cloned into pcDNA3.1/Myc-His B (Thermo Fisher Scientific). The Lrrc15 extracellular domain without a signal peptide (amino acids 22–536) was amplified by PCR using primers described in Table S1 and subcloned into pcDNA3.1/Myc-His B. Both a MycHis-tag and a Lrrc15 fragment were subcloned into pGEX-4T1 (Cytiva) using the *Bam*HI fragment-PmeI/*Sma*I restriction site. The Fantom3 clone of Dsg4 was purchased from Dnaform (FantomID_100066319), and the extracellular domain without a signal peptide (amino acids 50–632) was PCR-amplified using primers described in Table S1 and subcloned into pcDNA3.1/Myc-His B. A construct containing the MycHis-tag in frame with Dsg4 was PCR-amplified and subcloned into pGEX-4T1 using the *Bam*HI fragment-PmeI restriction site. Full-length Prdx6 (GeneBank accession no. NM_007453.4) and Sprr1b (GeneBank accession no. NM_009265.3) were PCR-amplified from mouse femur cDNA using primers described in Table S1 and subcloned into pGEX-4T1. Expression of recombinant glutathione S-transferase (GST) fusion proteins was induced in *Escherichia coli* Rosetta-gami B (Merck) using isopropyl β-d-thiogalactopyranoside (IPTG), which were then lysed in lysis buffer (40 mM *N*-(2-hydroxyethyl)piperazine-*N*′-ethanesulfonic acid (Hepes), 250 mM NaCl, 2 mM ethylenediaminetetraacetic acid (EDTA), 2 mM dithiothreitol (DTT), and 20 wt % sucrose) plus sonication. Recombinant proteins were solubilized in 0.1 wt % Triton X-100 and purified using glutathione-Sepharose 4B (Cytiva). To verify the purity and amount of recombinant proteins, a portion of purified proteins was separated by sodium dodecyl sulfate/polyacrylamide gel electrophoresis (SDS/PAGE) and stained with FastGene Q-Stain (Nippon Genetics).

**Figure 2:**
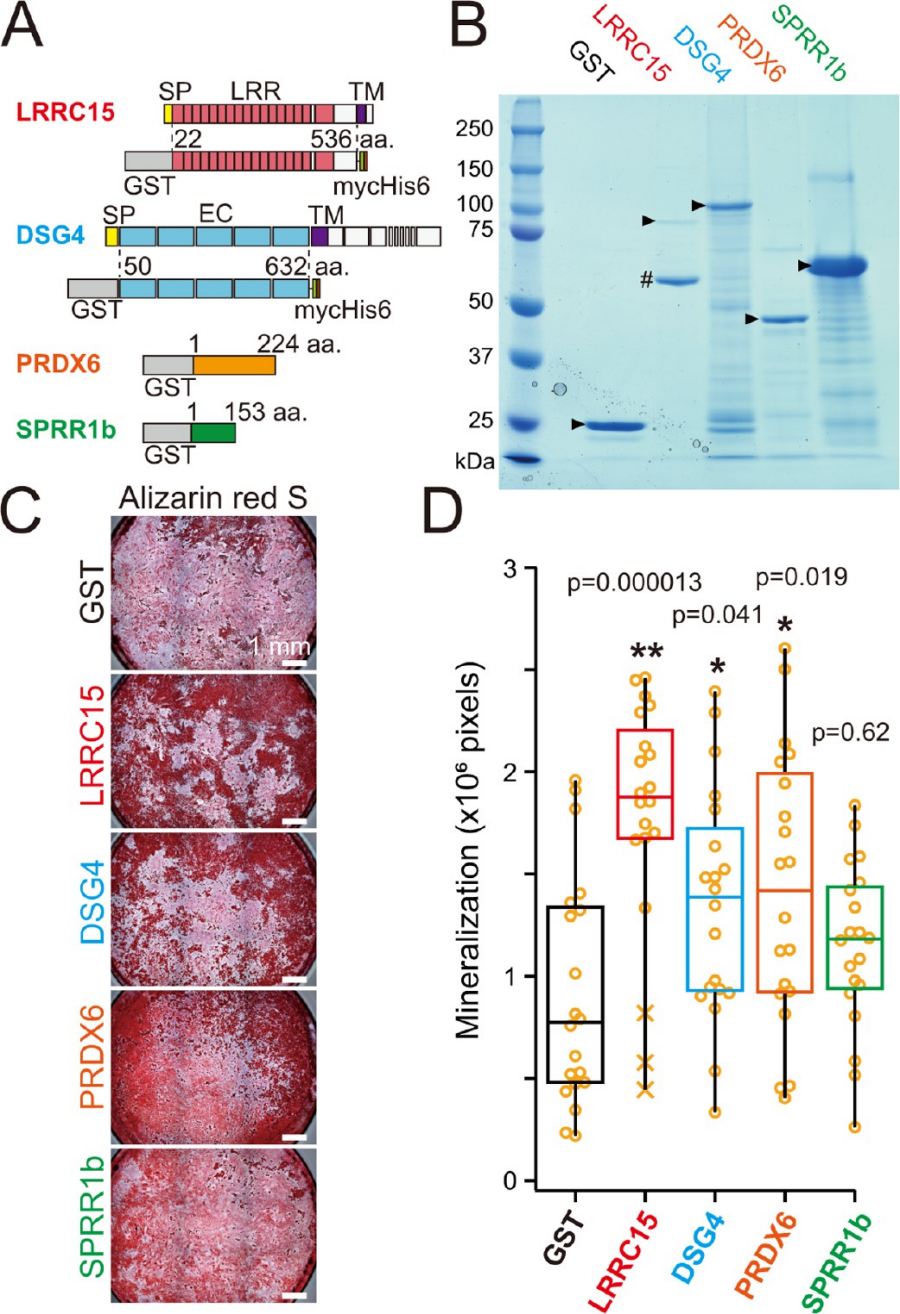
Bioactivity of recombinant mouse homologues in a mineralization assay. (A) Schematic showing candidate GST-fusion proteins. SP, signal peptide; LRR, leucine-rich repeat; TM, transmembrane region; EC, extracellular cadherin domain; mycHis6, myc and 6× histidine tag; aa, amino acids. (B) SDS-PAGE and CBB staining of purified GST-fusion proteins. Arrowheads, presumed full-length recombinant proteins. #, partially degraded protein. (C) Alizarin red S staining after a mineralization assay using calvarial osteoblasts cultured on dishes coated with candidate GST-fusion proteins or a GST control (1.68 μg/cm^2^). Dishes were stained 13 days after induction of osteoblast differentiation. Scale, 1 mm. (D) Quantification of mineralization assay shown in (C). Alizarin red S-positive areas were quantified in 10 random ROIs (1235 μm × 926 μm, pixel area: 0.415 μm^2^) per dish (duplicates) for each coating and are plotted as open circles. X, outlier. *p < 0.05, **p < 0.01.

Note that after purification of LRRC15 using the N-terminal GST, a partially degraded protein remained (Figure 2B), which was not removable by Ni-NTA agarose (Qiagen) targeting the C-terminal His-tag.

### Gene Expression Analysis Using Quantitative PCR

Primary calvarial osteoblasts were plated in 24-well plates coated with gelatin (0.1%, Thermo Fisher) and either GST or GST-fusion proteins (1.68 μg/cm^2^), and then cultured 4 days in osteoblast differentiation medium. Noninduced controls were similarly cultured in normal culture medium. At the end of the culture period total RNA was isolated using TRIzol LS reagent (Thermo Fisher) and a Direct-zol RNA Microprep Kit (Zymo Research), and cDNA was synthesized using ReverTra Ace qPCR Master Mix with gDNA Remover (Toyobo). Quantitative PCR analysis of genes of interest was conducted on a Viia7 real-time PCR system (Thermo Fisher) with Premix Ex Taq (Takara). Oligo Primers (Fasmac) and the Universal Probe Library (Roche) used for analysis are shown in Table S1.

### Amino Acid Conservation Analysis

Amino acid sequences of pig DSG4 (accession. no. XP_003356443.3), mouse DSG4 (accession. no. NP_853543.1), human DSG4 (accession. no. NP_001127925.1), pig PRDX6 (accession. no. NP_999573.1), mouse PRDX6 (accession. no. NP_031479.1), and human PRDX6 (accession. no. NP_004896.1) were obtained from GenBank, aligned, and the percent homology of recombinant protein region using this study (mouse DSG4 50–632, amino acids; mouse PRDX6, 1–224 amino acids) was calculated by counting the number of conserved amino acids using hierarchical clustering with MultiAlin (http://multalin.toulouse.inra.fr/multalin/).^11^

### Nanocomputed Tomography (Nano-CT)

The femur of a C57BL/6J mouse (P1) was mounted in a PCR tube to maintain humidity, and nano-CT images were obtained using a high-resolution three-dimensional (3D) X-ray microscope (nano3DX, Rigaku). The distal femur was scanned by 17 keV X-rays from a Mo-target anode (50 kV, 24 mA) with a 2048 × 2048 (6.5 μm/pixel) sCMOS detector (Zyla 4.2). A total of 600 projections was collected per 180° with a pixel size of 1.25 μm (L0540 lens, bin 2, exposure time, 15 s, FOV: 1.331 × 1.331 mm^2^) or 0.62 μm (L0270 lens, bin 2, exposure time, 20 s, FOV: 0.666 × 0.666 mm^2^) in step-scan mode with a 5 mm sample-to-detector distance. A PDMS micropattern chip was mounted onto an aluminum bar with a 3 mm diameter using double-sided tape. PDMS was scanned by 8 keV X-rays from a Cu-target anode (40 kV, 30 mA) with a 2048 × 2048 (6.5 μm/pixel) sCMOS detector (Zyla 4.2). A total of 200 projections was collected per 180° with a pixel size of 1.25 μm (L0270 lens, bin 4, exposure time, 4 s, FOV: 0.666 × 0.666 mm^2^) in continuous-scan mode with a 5 mm sample-to-detector distance. Data were analyzed using TRI/3DBON (FCS64, Ratock System Engineering) and 3D Slicer.^12^

### Micropatterning

Micropatterns (30 or 50 μm basic units) were designed using Photoshop (Adobe) and AutoCAD software (Autodesk). A glass mask for photolithography was made by contract manufacturing at Tokyo Metropolitan Industrial Technology Research Institute (TIRI). An SU-8 (Nippon Kayaku) mold was made on a silicon substrate using a spin coater (ACT-300A II, ACTIVE) and mask aligner (PEM-800, Union Optical). Fluorosurf (FG-5084SH-0-1, Fluorotech) was used as the releasing agent. Micropatterns were transferred to PDMS (Sylgard-184, DOW Chemical Company, 10:1 mix ratio). After washing with water, the PDMS test chip was dried and hydrophilized by plasma treatment using a PIB-10 vacuum device. The PDMS test chip was then coated with 0.05% type I atelocollagen (IPC-30, Koken) and, after PBS washing, coated with purified protein (100 ng/μL).

### Immunostaining

The culture bottom of an eight-well chamber slide was coated with a mixture of gelatin (0.1%, Thermo Fisher) and either GST or GST-fusion proteins (1.68 μg/cm^2^). Primary calvarial osteoblasts were cultured for 5 h on the chamber slide in osteoblast differentiation medium, fixed for 10 min in 4% Paraformaldehyde (PFA)/PBS, and permeabilized for 5 min in 0.1% Triton X-100/PBS. After blocking 1 h in a mixture containing 5% normal donkey serum, 10 μg/mL normal donkey immunoglobulin G (IgG), and 1% bovine serum albumin (BSA)/PBS, cells were incubated with rat anti-Runx2 (MAB2006, R&D systems, 5 μg/mL) and either rabbit anti-alkaline phosphatase antibody (NBP2-67295, Novus Biologicals, 5 μg/mL) and mouse anti-phospho-SAPK/JNK antibody (#9255, Cell Signaling, 4 μg/mL), or rabbit anti-phospho-p38 MAPK (#9211, Cell Signaling, 5 μg/mL) and mouse anti-phospho-p44/42 MAPK antibody (#9106, Cell Signaling, 4 μg/mL) at 4 °C overnight. After three PBS washes (5 min each), cells were incubated with Alexa fluor plus 555 donkey anti-rabbit IgG (A32794, Thermo Fisher, 1:1000), Alexa fluor plus 647 donkey anti-mouse IgG (A32787, Thermo Fisher, 1:1000) and Alexa fluor plus 488 donkey anti-rat IgG (A48269TR, Thermo Fisher, 1:1000) at RT for 1 h. After three PBS washes (5 min each), cells were mounted in ProLong glass (Thermo Fisher) and photographed using Thunder Imaging Systems. Quantitation was performed using ImageJ (NIH). MC3T3-E1 cells were cultured for 4 days on a PDMS chip coated with type I aterocollagen. After PBS washing, cells were fixed for 10 min in 4% PFA/PBS and permeabilized for 5 min in 0.1% Triton X-100/PBS. After blocking 1 h in a mixture containing 5% normal donkey serum, 10 μg/mL normal donkey IgG, and 1% BSA/PBS, cells were incubated with rat anti-ZO-1 antibody (R26.4C, Developmental Studies Hybridoma Bank, 2 μg/mL) and mouse anti-β catenin antibody (610153, BD Biosciences, 2 μg/mL) at 4 °C overnight. After three PBS washes (5 min each), cells were incubated with Alexa fluor plus 555 donkey anti-rat IgG (A48270, Thermo Fisher, 1:1000), Alexa fluor plus 488 donkey anti-mouse IgG (A32766, Thermo Fisher, 1:1000), Alexa fluor plus 647 Phalloidin (A30107, Thermo Fisher, 1:500) and 4′,6-diamidino-2-phenylindole (DAPI) (D9542, Sigma-Aldrich, 2 μg/mL) at RT for 1 h. After three PBS washes (5 min each), cells were mounted in ProLong glass (Thermo Fisher). Images were obtained by confocal laser scanning microscopy (FV3000, Olympus).

### Immunohistochemistry

For confocal microscopy imaging, *Col1a1*-AcGFP mouse or *TRAP*-tdTomato mouse femur was fixed in 2% PFA/PBS overnight at 4 °C, embedded and sectioned at 10 μm using Kawamoto’s film method.^13^ Frozen femur sections were permeabilized with 0.2% Tween20/PBS and blocked in 5% normal donkey serum, 10 μg/mL normal donkey IgG, and 1% BSA/PBS. Sections were incubated overnight at 4 °C with primary antibodies: either rabbit anti-DSG4 (ab230787, abcam, 10 μg/mL) or rabbit anti-PRDX6 (ab59543, abcam, 1/50) and goat anti-MMP9 (AF909, R&D, 1:160). Sections were washed in PBS and incubated with secondary antibodies: Alexa Fluor 647 donkey anti-rabbit IgG (A31573, Thermo Fisher, 1:300) and Alexa Fluor 568 donkey anti-goat IgG (A11057, Thermo Fisher, 1:500) and stained with 1 μg/mL DAPI (D9542, Sigma-Aldrich, 2 μg/mL). Images were obtained using a confocal laser scanning microscope (FV3000, Olympus).

### Timelapse Imaging

GFP-MC3T3-E1 cells were seeded on PDMS micropatterns in a 3.5 cm dish at 1.5 × 10^5^ cells/dish. After 24 h, the culture medium was changed to osteoblast differentiation medium containing 50 μg/mL magnesium l-ascorbate/10 mM disodium β-glycerophosphate without phenol red. Live cell imaging was performed with the stage top incubator (Tokai hit) and a Nikon C2 confocal laser scanning microscope (10× objective lens, interval 1 h, total 50 h, *Z* = 15, *Z* step 11.3 μm). Image analysis and cell tracking were performed using IMARIS software (Bitplane).

### Statistics

Statistical comparisons between two independent groups of data were performed using Student’s *t* test. Other statistical analysis was conducted using IgorPro 9.0 software (HULINKS). Outliers were determined using Tukey’s inner fences.

## Results

### Identification of Bioactive Proteins That Enhance Osteogenesis

We hypothesized that calcified cartilage digested by chondroclasts (which are similar to osteoclasts) contains substances that serve as excellent scaffolds for bone formation, and thus prepared a crude extract from the pig femur growth plate, which contains abundant calcified cartilage. To mimic chondroclastic digestion, we treated freeze-fractured calcified cartilage with pepsin in a solution of 0.01 N hydrochloric acid to extract extracellular matrix from porcine articular cartilage.^14^ We then coated the bottom of a culture dish with that extract and performed a mineralization assay using an osteoblast cell line. As controls, we also prepared extract without pepsin. Control extracts did not promote mineralization, but the same extract with pepsin significantly promoted mineralization *in vitro* (Figure 1A). We then fractionated the pepsin-treated extract by centrifugation and selected a bioactive fraction (S4) based on the mineralization activity (Figures 1B and S1A). Fraction S4 was further fractionated into 45 subfractions by gel filtration chromatography, which we then evaluated in the mineralization assay (Figure S1B). Four fractions promoted mineralization. Proteins contained in a pooled sample of these fractions were separated by electrophoresis and silver staining, and candidate osteogenic molecules identified by mass spectrometry, revealing four candidate porcine proteins, namely, Leucine Rich Repeat Containing Protein 15 (LRRC15), Desmoglein 4 (DSG4), Peroxiredoxin 6 (PRDX6), and Small Proline Rich Protein 1B (SPRR1b).

We then cloned murine homologues of the four candidate porcine molecules, expressed them in bacteria, and purified them as GST-fusion proteins (Figure 2A,B). We coated culture dish surfaces with type I atelocollagen and each GST-fusion protein or GST control and conducted a mineralization assay using mouse primary calvarial osteoblasts (Figure 2C). Three candidates (LRRC15, DSG4, and PRDX6) significantly promoted *in vitro* mineralization by osteoblasts (Figure 2D).

**Figure 3:**
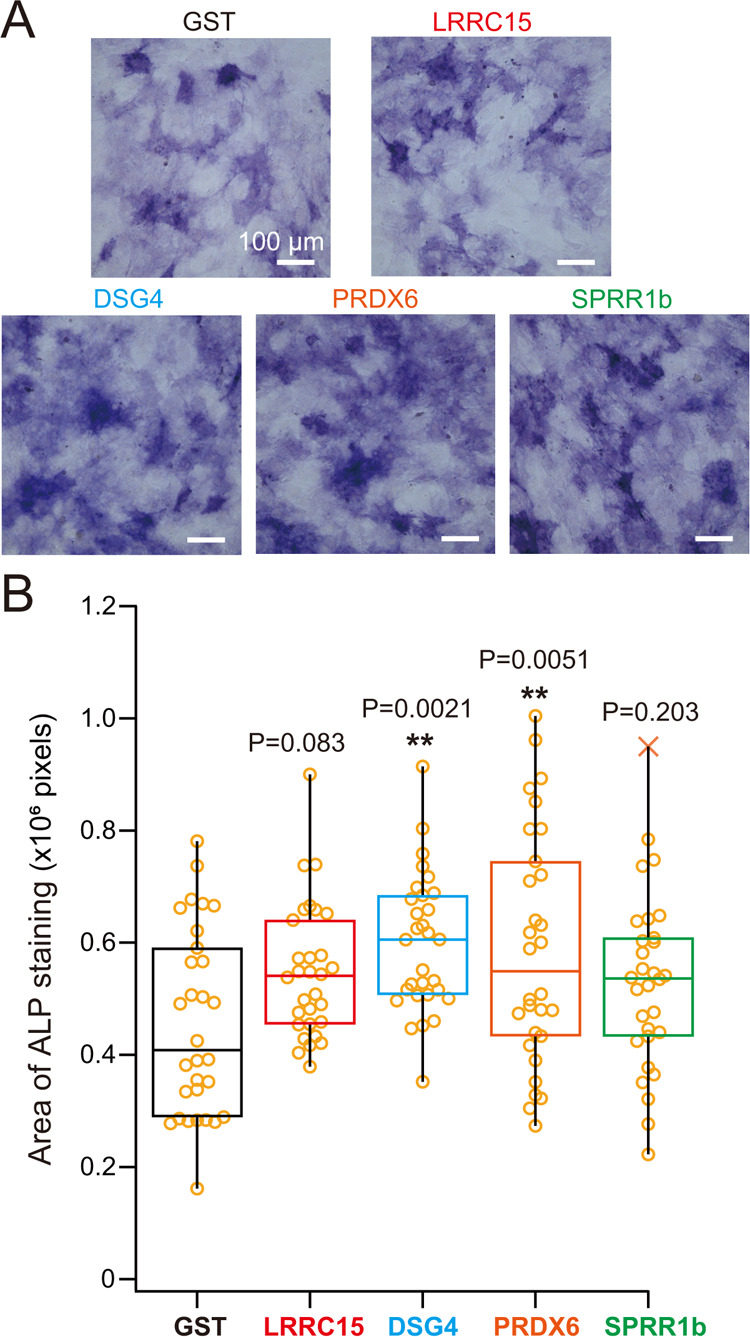
Effect of recombinant murine candidate proteins on osteoblast differentiation. (A) ALP activity staining of osteoblast differentiation using calvarial osteoblasts cultured on dishes coated with indicated GST-fusion proteins or a GST control (1.68 μg/cm^2^). Cells were stained 4 days after induction of osteoblast differentiation. (B) Quantification of ALP activity shown in (A). Data from each area (659 μm × 659 μm, pixel area: 0.415 μm^2^) is plotted as open circles. X, outlier; *N* = 30; three independent experiments. **p < 0.01.

We also examined the effects on osteoblast differentiation by seeding calvarial osteoblasts on culture dishes coated with each of the four candidate molecules and then staining with ALP, a marker of early-stage osteoblast differentiation. Based on that analysis, two candidates (DSG4 and PRDX6) significantly promoted osteoblast differentiation (Figure 3). DSG4 and PRDX6 enhanced osteoblast differentiation concentration-dependently (Figure S2A). Moreover, DSG4 also significantly increased *Bglap* (late-stage osteoblast marker) expression, and PRDX6 increased *Alp1* (early-stage osteoblast marker) and *Bglap* expression relative to control GST after 4 days of osteoblast differentiation (Figure S2B). By contrast, the expression of *Col1a1*, which is widely expressed in osteoblastic lineage cells, was not altered by any treatment.

When we examined DSG4 and PRDX6 amino acid conservation across species, we found that recombinant protein regions of DSG4 exhibited >81% homology among pig, mouse, and human species. Similarly, PRDX6 exhibited 90% homology (Figure S3). Thus, given their stimulatory effects on bone formation and osteoblast differentiation, we focused on DSG4 and PRDX6 in the remainder of this study.

**Figure 4:**
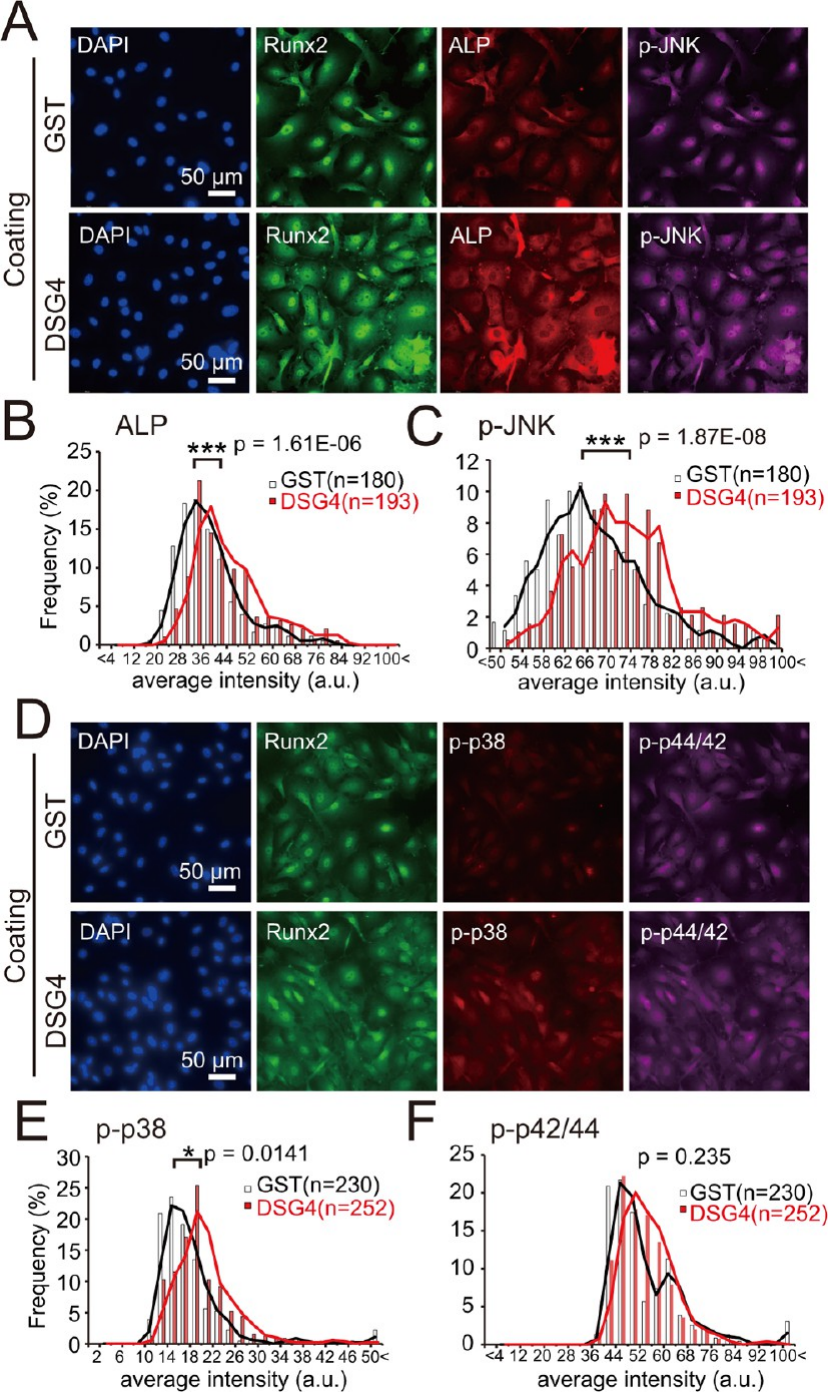
Culturing of calvarial osteoblasts on DSG4-coated dishes enhances MAPK signaling. (A) Immunocytochemistry of Runx2, ALP, and phosphorylated JNK (p-JNK) in calvarial osteoblasts cultured on dishes coated with GST or GST-DSG4. Image was acquired 5 h after start of culture in osteoblast differentiation media. Scale, 50 μm. Quantification of ALP expression (B) and JNK phosphorylation (C) shown in (A). Data is representative of three independent experiments. Total cell numbers are indicated in each graph. ***p < 0.001. (D) Immunocytochemistry of Runx2, phosphorylated-p38 MAPK (p-p38), and phosphorylated p44/42 MAPKs (p-p44/42) in calvarial osteoblasts cultured on dishes coated with GST or GST-DSG4. Images were acquired 5 h after start of culture in osteoblast differentiation media. Scale, 50 μm. Quantification of p38 MAPK (E) and p44/42 MAPK (F) phosphorylation shown in (D). Data are representative of three independent experiments. Total cell numbers are indicated in each graph. *p < 0.05.

We next investigated signaling mediating osteoblastic differentiation. p38 mitogen-activated protein kinase (MAPK) and c-Jun-NH2-terminal kinase (JNK) pathways are activated during BMP-2-induced osteoblastic differentiation.^15^*Dsg4* belongs to a gene family encoding desmosomal cadherins, grouped as desmogleins (*Dsg1*–*Dsg4*) and desmocollins (*Dsc1*–*Dsc3*).^16^ Curiously, DSG2, in addition to its adhesion function, regulates intestinal barrier function *via* p38 MAPK signaling.^17^ Thus, we analyzed MAPK signaling in Runx2-positive calvarial osteoblasts cultured on DSG4-coated dishes (Figure 4A). Consistent with enhanced ALP activity seen in calvarial osteoblasts cultured on DSG4 (Figure 3), ALP expression increased in osteoblasts cultured on DSG4 (Figure 4B). Moreover, osteoblasts cultured on DSG4 exhibited enhanced JNK (Figure 4C) and p38 MAPK (Figure 4D,E) phosphorylation, but p42/44 Erk phosphorylation was unchanged (Figure 4F). These data suggest that DSG4 activates JNK and p38 MAPK signaling, which may enhance osteoblast differentiation.

**Figure 5:**
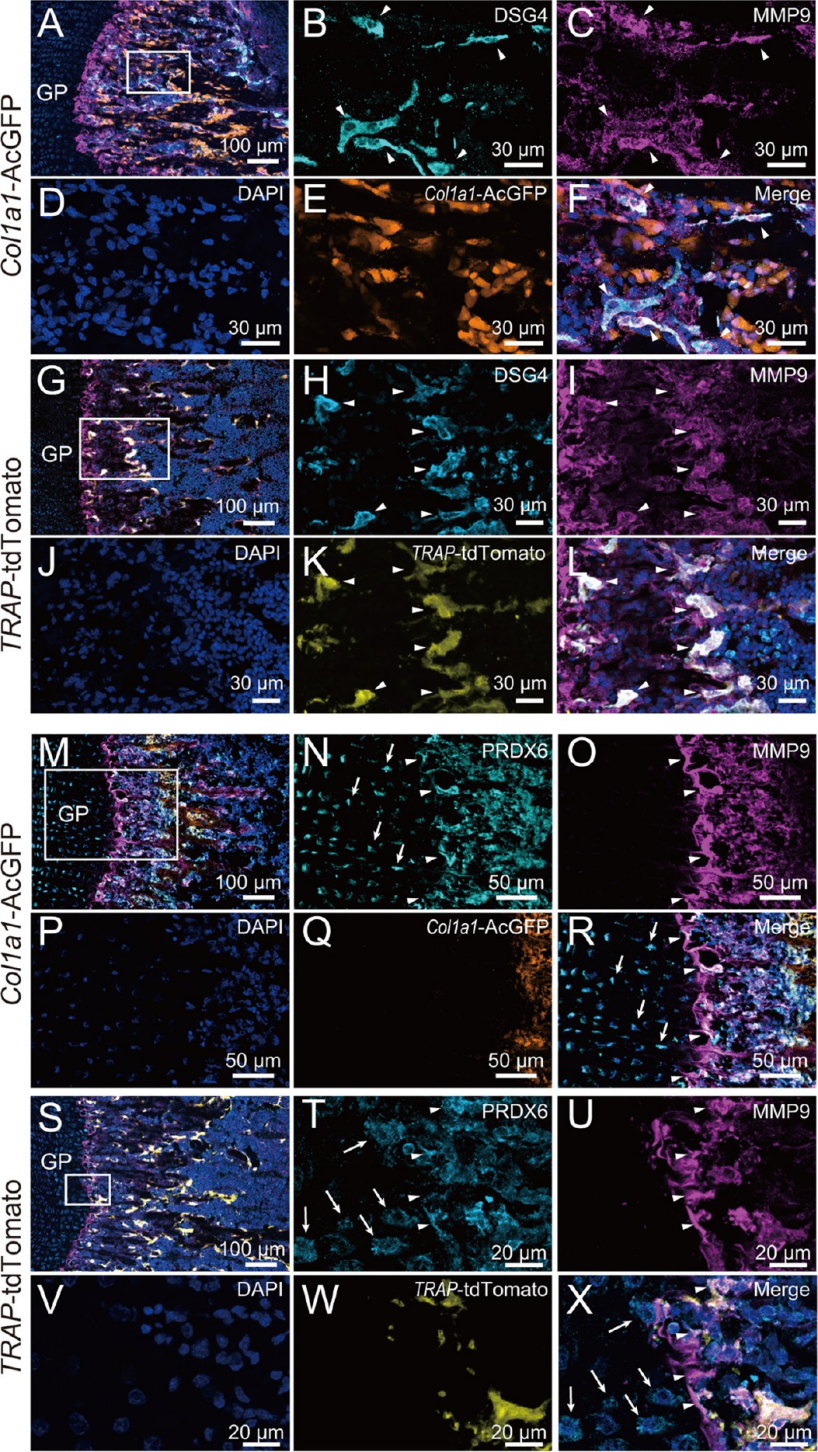
DSG4 and PRDX6 expression in mouse femur. (A) Merge of immunohistochemistry of *Col1a1*-AcGFP mouse femur metaphysis at P22. Shown is staining for DSG4 (cyan), MMP9 (magenta), DAPI (blue), and AcGFP (orange). GP, growth plate. Scale, 100 μm. (B–F) Magnified views of boxed area in (A). Arrowheads, MMP9-positive osteoclasts expressing DSG4. Scale, 30 μm. (G) Merge of immunohistochemistry of *TRAP*-tdTomato mouse femur metaphysis at P28. Shown is staining for DSG4 (cyan), MMP9 (magenta), DAPI (blue), and tdTomato (yellow). GP, growth plate. Scale, 100 μm. (H–L) Magnified views of boxed area in (G). Arrowheads, *TRAP*-tdTomato-positive osteoclasts expressing DSG4. Scale, 30 μm. (M) Merge of immunohistochemistry of *Col1a1*-GFP mouse femur metaphysis at P22. Shown is staining for PRDX6 (cyan), MMP9 (magenta), DAPI (blue), and AcGFP (orange). GP, growth plate. Scale, 100 μm. (N–R) Magnified views of boxed area in (M). Arrows, PRDX6-positive chondrocytes; arrowheads, MMP9-positive chondro-osseous junction showing PRDX6. Scale, 50 μm. (S) Merge of immunohistochemistry of *TRAP*-tdTomato mouse femur metaphysis at P28. Shown is staining for PRDX6 (cyan), MMP9 (magenta), DAPI (blue), and tdTomato (yellow). GP, growth plate. Scale, 100 μm. (T–X) Magnified views of boxed area in (S). Arrows, PRDX6-positive chondrocytes; arrowheads, *TRAP*-tdTomato-negative and MMP9-positive chondro-osseous junction showing PRDX6. Scale, 30 μm.

To determine whether the porcine osteogenic candidates DSG4 and PRDX6 are also expressed in mouse femur and assess their potential contribution to endochondral ossification, we performed immunohistochemistry for both proteins in the femoral growth plate of P22 to P28 mice. This period is when osteoblasts actively form bone on calcified cartilage. Specifically, we used the femur of *Col1a1*-AcGFP mice to visualize osteoblasts and stained osteoclasts with MMP9 antibody. As shown in Figure 5A, we observed a DSG4 immunosignal in the metaphysis of the femur. Higher-magnification images revealed DSG4 protein expression in MMP9-positive osteoclasts (Figure 5B,C). We also observed *Col1a1*-positive osteoblasts in the vicinity of these osteoclasts (Figure 5D–F). In the chondro-osseous junction, MMP9 immunosignals were detected both in osteoclasts and partially absorbed mineralized cartilage. To distinguish between osteoclasts and cartilage, we next performed staining of the femur of *TRAP*-tdTomato mice (Figure 5G) and observed DSG4 expression in *TRAP*-tdTomato-positive osteoclasts (Figure 5H–L). These findings suggest that DSG4 derived from osteoclasts may activate osteoblast-lineage cells.

On the other hand, we observed extensive PRDX6 expression in the distal femur (Figure 5M). Higher-magnification images showed PRDX6 immunosignals in growth plate chondrocytes (Figure 5N, arrows) and in MMP9-positive osteoclasts and partially absorbed mineralized cartilage (Figure 5O, arrowheads). *Col1a1*-positive osteoblasts appear only on primary trabecular bone toward the diaphysis (Figure 5P–R). In the chondro-osseous junction of TRAP-tdTomato mice (Figure 5S), we observed high PRDX6 expression in chondrocytes and in TRAP-toTomato-negative, MMP9-positive, partially absorbed mineralized cartilage (Figure 5T–X). These observations suggest that PRDX6 is present in calcified cartilage and serves as a scaffold for bone formation by osteoblasts, following absorption by chondroclasts.

### Identification of Surface Shapes Favoring Mineralization

**Figure 6:**
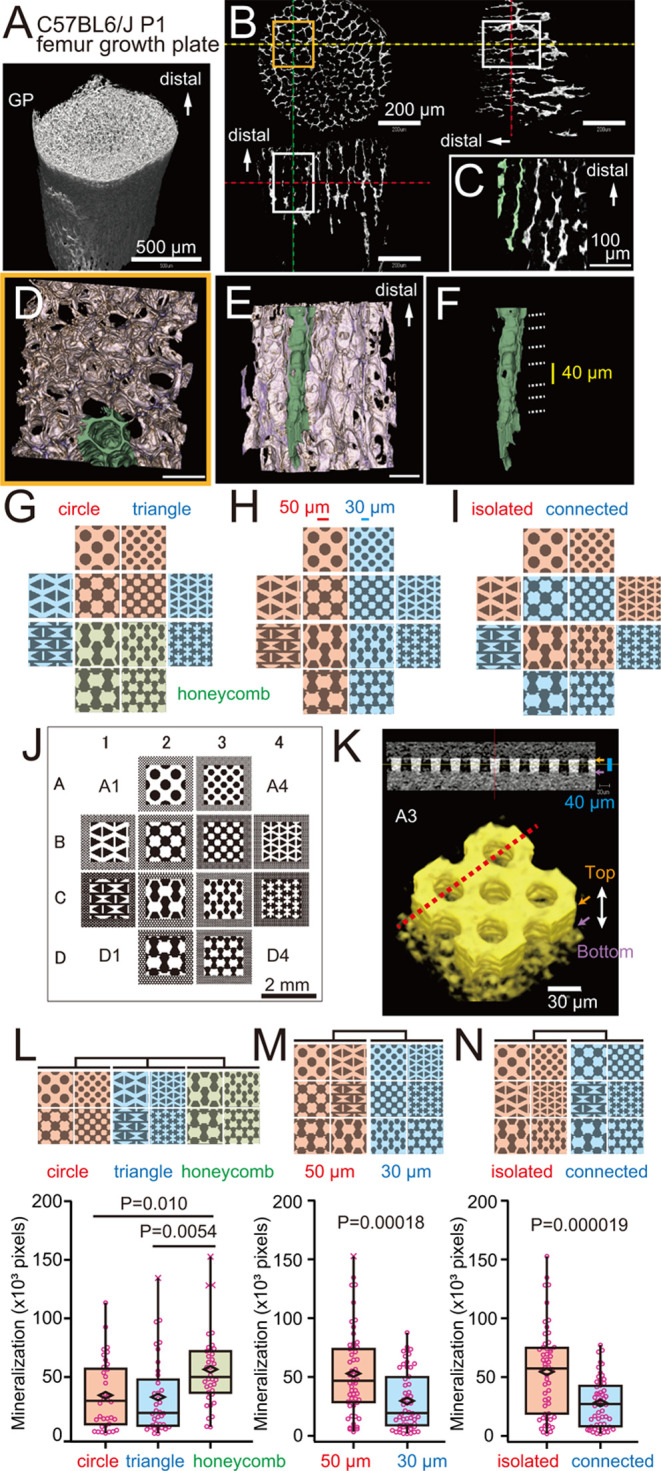
Structure of osteogenic surface on calcified cartilage of mouse femur and design of micropatterns. (A) Overview of nano-CT image of calcified cartilage and bone of P1 mouse femur. GP, growth plate; scale, 500 μm. (B) Slice of 2D image from nano-CT image of calcified cartilage in the metaphysis. Extracted areas analyzed in (C)–(F) are indicated by boxes. Scale, 200 μm. (C) Representative 2D image of calcified cartilage. A part of the longitudinal septa is pseudocolored green and further analyzed in (D)–(F). Scale, 100 μm. (D) Cross-sectional view of polygonal septa in calcified cartilage. Scale, 50 μm. (E) Longitudinal view of polygonal septa in calcified cartilage. Scale, 50 μm. (F) Polygonal septa exhibit an array of osteogenic compartments regularly spaced by ∼40 μm (dotted lines). (G–I) Schematics showing micropattern shapes. Differences in micropattern shape (G), size (H), and connectivity (I) are highlighted by distinct colors. (J) Overview of designed micropatterns. Magnified patterns are superimposed on original micropatterns. Scale, 2 mm. White, top surface (protruded). Black, bottom surface (retracted) of micropatterns. (K) Nano-CT image of PDMS micropattern A3 shown in (J). Slice image of the red dotted line is shown above. White scale, 30 μm; blue scale, 40 μm. (L–N) Osteogenic activity of calvarial osteoblasts on various PDMS micropatterns, as indicated by area of Alizarin red S staining (pixel area: 2.59 μm^2^). (L) Mineralization on circle (A2, A3, B2 and B3), triangle (B1, B4, C1 and C4), or honeycomb (C2, C3, D2 and D3) micropatterns coated with control GST. (M) Mineralization on 50 μm (A2, B1, B2, C1, C2, and D2) or 30 μm (A3, B3, B4, C3, C4, and D3) micropatterns coated with control GST. (N) Mineralization on isolated (A2, A3, B1, B4, C2, and C3) or connected (B2, B3, C1, C4, D2, and D3) micropatterns coated with control GST.

Calcified cartilage located directly beneath the proximal growth plate of the femur is characterized by multiple compartment-like structures derived from hypertrophic chondrocytes (Figure 6A). We hypothesized that this characteristic structure contributes to initiation of bone formation since bone formation by osteoblasts progresses on a scaffold of calcified cartilage. To confirm this activity, we first performed imaging of femoral calcified cartilage beneath the growth plate of P1 mice using high-resolution X-ray CT. A cross-section of longitudinal septa of calcified cartilage showed a polyhedral mesh-like pattern (Figure 6B). We also observed a longitudinal arrangement of delimited spaces inside the columns of stacked hypertrophic chondrocytes along the intercolumnar septa (Figure 6C). After physiological death of hypertrophic chondrocytes (chondroptosis) or their trans-differentiation into osteoblasts, the surface of the intercolumnar septa becomes osteogenic scaffold^18^ (Figure 6D,E). Three-dimensional-rendered images further revealed such surfaces consisted of repeating compartments of about 40 μm along the intercolumnar septa (Figure 6F).

Based on these findings, we assessed the impact of shape, size, and connectivity on bone formation, by testing various combinations of these three elements. To do so, we designed 12 micropatterns by simplifying the complex shape of calcified cartilage to circle, triangle, or honeycomb (Figure 6G), of large or small size (50 or 30 μm units) (Figure 6H), and in isolated or connected arrangements (Figure 6I). We arranged the 12 plus four control flat surfaces in a 4 × 4 pattern (Figure 6J). Micropatterns were transferred onto poly(dimethylsiloxane) (PDMS) from the SU-8 master mold. To minimize the influence of neighboring patterns, we cut out three different subregions from a PDMS sheet containing the 4 × 4 pattern >50 times and used them for mineralization assays (Figure S4). To confirm micropattern height, we performed nano-CT imaging of the PDMS micropattern at position A3, as seen in Figure 6J, and found the height of PDMS micropatterns to be ∼40 μm (Figure 6K).

We then rendered the PDMS surface hydrophilic by plasma treatment, coated micropatterns with type I atelocollagen, and then with DSG4-GST, PRDX6-GST, or control GST, performed a mineralization assay using mouse calvarial osteoblasts, and stained mineralized nodules with alizarin red S. Positively staining areas were quantified relative to control GST-coated samples. Analysis revealed that the honeycomb structure significantly promoted mineralization relative to circles and triangles (Figure 6L). Moreover, relevant to size, we observed a significant promotion of mineralization using larger rather than smaller patterns (Figure 6M). Finally, when we analyzed isolated *versus* connected units, mineralization was significantly enhanced in micropatterns with isolated basic units (Figure 6N).

### Micropattern Effects on Osteoblast Migration

**Figure 7:**
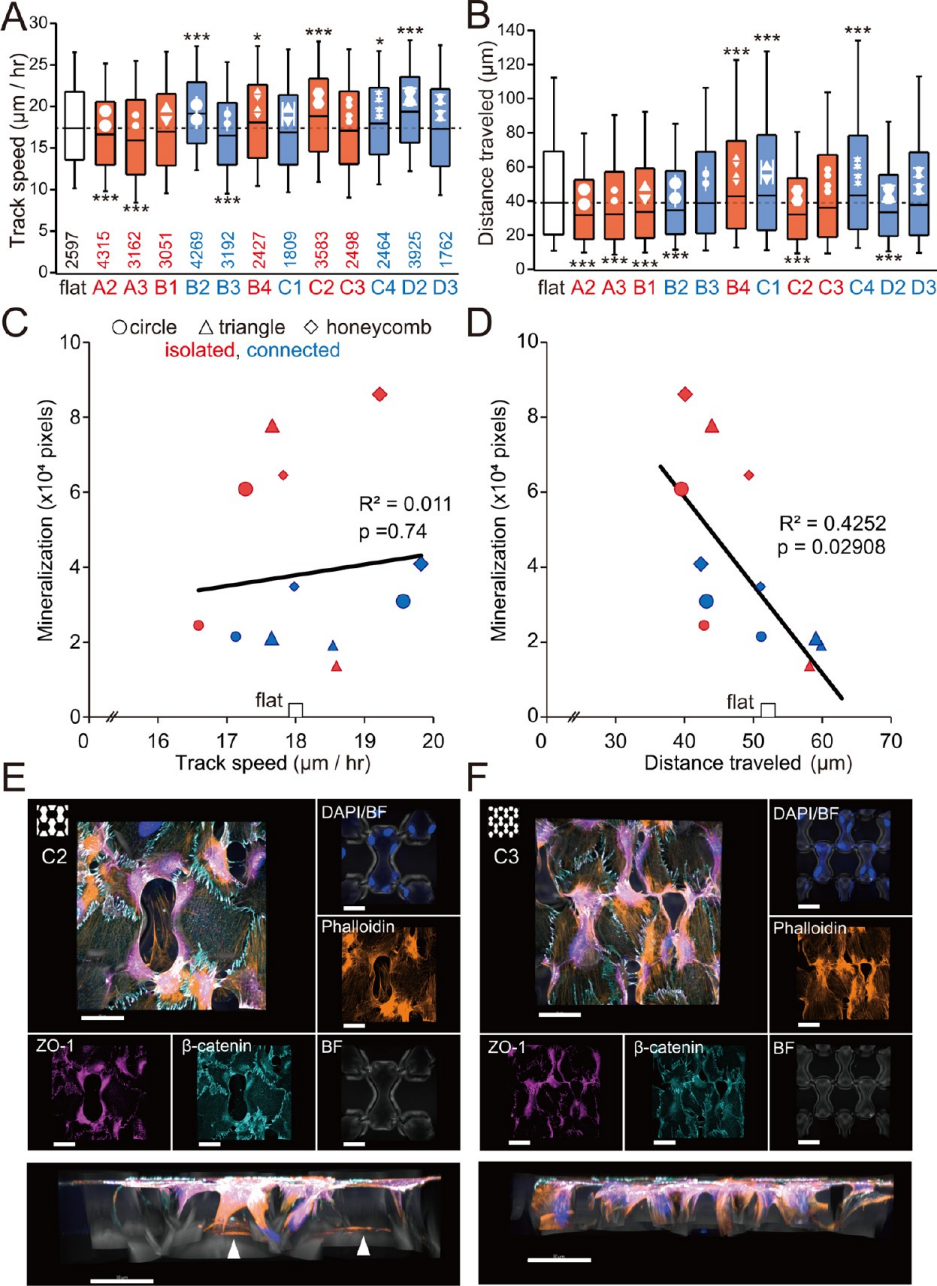
Tracking analysis of GFP-MC3T3-E1 cells on PDMS micropatterns. (A) Average speed of cells moving on indicated micropatterns. *p < 0.05, ***p < 0.01. Total number of cells tracked is indicated in the graphs. (B) Distance traveled from the start of tracking for cells moving on indicated micropatterns. ***p < 0.01. Correlation of track speed (C) (as in (A)) and distance traveled (D) (as in (B)) with average mineralization (Figure 6L–N). (E) Immunocytochemistry of MC3T3-E1 cells cultured on large honeycomb micropatterns for cell adhesion marker antibodies (β-catenin and ZO-1) and phalloidin (for cell morphology). BF, bright field (differential interference contrast). Top panels: top view; bottom panel, side view. In the bottom panel, note that phalloidin-positive cells enter a gap in the micropattern and become attached to the bottom of the pattern (arrowheads). (F) Comparable immunocytochemistry of MC3T3-E1 cells cultured on the small honeycomb micropattern. Top panels, top view; bottom panel, side view. Scale, 30 μm.

Osteoblasts and their progeny migrate within a three-dimensional bone space to reach sites of bone formation.^19^ Osteogenesis can be promoted by influencing the migration of osteoblasts. To understand how micropattern shapes affect bone formation by osteoblasts, we therefore analyzed cell migration on micropatterns using live cell imaging of GFP-MC3T3-E1 cells. Specifically, we measured mean migration speed (Figure 7A) and migration distance from the starting point of tracking (Figure 7B) on each micropattern and found that different micropatterns were associated with variations in migration speed and distance. We then correlated mineralization on each micropattern measured in Figure 6L–N with either mean migration speed (Figure 7C) or distance (Figure 7D). Interestingly, mineralization on all micropatterns was negatively correlated with migration distance. To further analyze this outcome, we performed immunostaining of MC3T3-E1 cells cultured on large (C2) and small (C3) honeycomb micropatterns for cell adhesion marker antibodies (β-catenin and ZO-1) and phalloidin (for cell morphology). We observed cells that appeared to have partially entered honeycomb holes, while some cells fully adhered to the bottom surface of large, but not small, micropatterns (Figure 7E,F), suggesting that cells resided in the large micropattern and that their migration was restricted.

### Combinatorial Effects of Coat Proteins and Micropatterns

**Figure 8:**
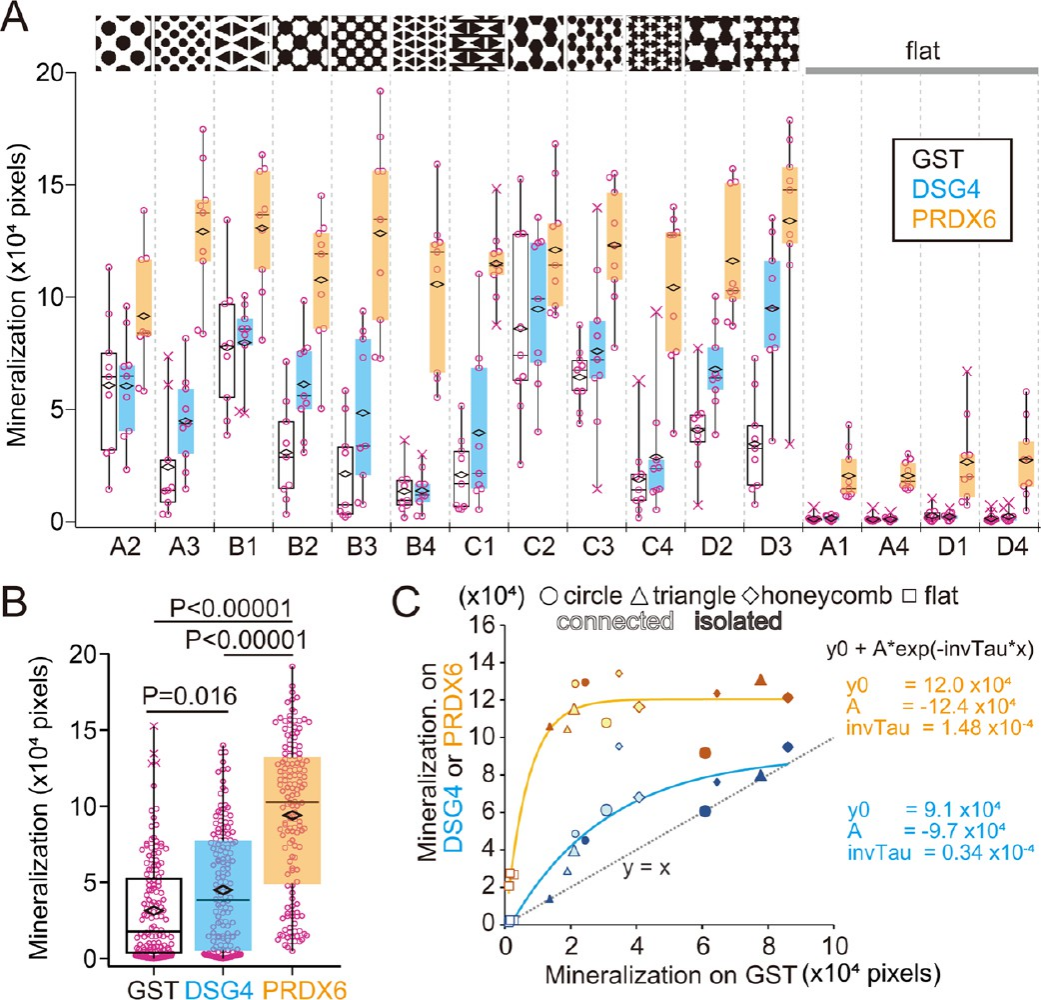
Osteogenic activity of calvarial osteoblasts cultured on PDMS micropatterns coated with GST, GST-DSG4, or GST-PRDX6. (A) Quantification of mineralization based on the area of Alizarin red S staining (pixel area: 2.59 μm^2^) on indicated micropatterns, as shown in Figure 6J. Triplicate analysis is shown of three different arrangements shown in Figure S4. Diamonds in box plots indicate average values (nine data points). X, outlier. (B) Quantification of mineralization on all patterns coated as indicated (144 data points each). (C) Correlation of average mineralization on GST only on each micropattern with that on and DSG4- or PRDX6-coated patterns. Open squares indicate flat surfaces (A1, A4, D1, and D4). Large and small markers indicate 50 and 30 μm micropatterns. Circle, triangle, and diamond markers indicate corresponding micropatterns. Filled and pastel markers indicate isolated and connected micropatterns, respectively. Data were fitted using Igor software with an exponential curve, *y*_0_ (initial value) estimates the maximum mineralization on DSG4- or PRDX6-coated patterns, and *A* (amplitude) and InvTau (decay rate) estimate the enhancement of mineralization by micropattern with coating.

Finally, to analyze the possible combinatorial effects of DSG4 or PRDX6 activity with surface geometry, we coated the type I atelocollagen-precoated PDMS micropatterns with either GST-fusion DSG4 or PRDX6 proteins or control GST and conducted mineralization assays using mouse calvarial osteoblasts. Mineralized nodules were alizarin red S-stained and the area of positive staining was quantified (Figure 8A). Consistent with the results seen in flat culture dishes (Figure 2), DSG4 or PRDX6 coating also promoted mineralization on PDMS micropattern on average of all patterns (Figure 8B). To assess a potential relationship between coating and micropattern geometry, we evaluated mineralization on each pattern with each of the coatings (Figure 8C). PRDX6 coating strongly enhanced osteogenic potentials of surfaces and could override geometrical cues, while DSG4-coating more moderately enhanced osteogenic potentials on top of the effect of each geometry. Overall, we observed an additive effect of DSG4 or PRDX6 coating with micropattern geometry.

## Discussion

Here, our goal was to mimic the osteogenic properties of calcified cartilage based on both the presence of osteogenic molecules and geometry. Specifically, using calcified cartilage as starting material, we searched for coating proteins that enhanced bone formation *in vitro* and also designed surface patterns based on structural analysis using high-resolution nano-CT images of calcified cartilage. We then evaluated the osteogenic effect of micropatterns combined with specific coatings.

Because samples of mouse calcified cartilage are too small to fractionate biochemically, we chose the growth plate of the porcine femur as the starting material and identified candidate osteogenic factors promoting bone formation *in vitro*, using crude extracts from pepsin-treated-calcified cartilage. We then cloned mouse homologues of porcine candidates and investigated their expression in mouse femur and osteogenic effects *in vitro*. We believe this strategy is valid given the cross-species conservation of genes encoding osteogenic factors. Based on enhanced *in vitro* mineralization and osteoblast differentiation seen in the presence of murine homologues, we judged DSG4 and PRDX6 to have osteogenic activity.

DSG4 is a type I cell surface cadherin family protein expressed in MMP9-positive osteoclasts in mice. It may enhance osteogenesis through direct interaction between osteoclasts and osteoblasts, a mechanism reminiscent of RANK/RANKL^20,21^ or ephrinB2/EphB4.^22^ Alternatively, the cleaved extracellular domain of DSG4 may contribute to osteoblast activation. Osteoclasts themselves reportedly secrete osteogenic factors,^23^ and indeed, we showed that the DSG4 extracellular domain enhances *in vitro* bone formation. Consistently, MMP9 and ADAM10 cleave cadherin ectodomains of DSG2 on intestinal epithelial cells, soluble DSG2 functions as a secondary signaling molecule,^24^ and MMP9 and ADAM10 are expressed in osteoclasts.^25,26^ DSG4 may function as an osteogenic molecule on the resorption surface during endochondral ossification. Prior to bone formation by osteoblasts, which progresses from the absorption surface,^27^ calcified cartilage is partially resorbed by osteoclasts. Through this process, the resorption surface may be coated with DSG4 extracellular domain protein released from osteoclasts.

PRDX6 is a member of the thiol-specific antioxidant protein family that can reduce hydrogen peroxide, short-chain organic fatty acids, and phospholipid hydroperoxides.^28^ We observed PRDX6 immunosignals in growth plate chondrocytes and in the MMP9-positive osteoclasts and partially absorbed mineralized cartilage. PRDX6 may contribute to bone formation by altering reduction–oxidation (redox) status in a manner that enhances osteogenesis. Consistently, continuous treatment with exogenous hydrogen peroxide reportedly inhibits osteogenic differentiation of human umbilical cord-derived MSCs cultured on polystyrene dishes but not on extracellular matrix.^29^ Extracellular redox states also alter adipogenesis in the mouse embryonic fibroblast line 3T3-L1: more reduced extracellular redox states inhibit adipogenesis, while more oxidizing conditions promote it.^30^ MSCs can differentiate into multiple lineages including osteogenic and adipogenic lineages. During osteogenesis, intracellular cysteine redox potentials decrease.^31^ As a peroxidase, PRDX6 promotes the reduction of target molecules. Chondrocytes express PRDX6, and PRDX6 derived from apoptotic chondrocytes may reduce disulfide bonds in extracellular matrix proteins of calcified cartilage and enhance osteoblast differentiation. A limitation of this study is that the effects of candidate osteogenic molecules on bone formation were assayed using calvarial osteoblast culture. However, analysis of in vivo models is necessary to devise therapeutic applications, such as osteogenic modification of implants. Thus, future studies should assess repair by intrinsic osteoblasts after insertion of implants with DSG4 and PRDX6-coated micropatterns into injury sites of mouse or pig long bones followed by an analysis of bone-forming ability.

We also designed micropatterns based on calcified cartilage structure and evaluated their effect on bone formation. When we analyzed differences between basic structural units, such as circles, triangles, and honeycomb patterns, the honeycomb structure significantly promoted mineralization relative to circles or triangles. Interestingly, higher activity in terms of honeycomb > circle > triangle may depend on the respective areas of these shapes: significant promotion of mineralization was consistently seen with larger rather than smaller patterns. Furthermore, mineralization was significantly promoted in micropatterns with isolated *versus* connected units, indicating that enclosed compartment is beneficial for bone formation.

We also showed that the large honeycomb patterns with high bone formation capacity contained osteoblasts fully adhering to the bottom surface of honeycomb holes. Consistently, others have reported that cells on osteogenic topographies were confined between structures.^32^ On micropatterns, mineralization was inversely correlated with the distance of cell movement, rather than cell migration speed. Micropatterns reduce the linearity of cell movement, analogous to walking through a maze. Thus, osteoblasts repeatedly pass through the same location, and relative dwell time in micropatterns is likely prolonged compared to that on a flat surface. In endochondral ossification, calcified cartilage forms an osteogenic compartment, which may promote bone formation by restricting osteoblast movement. These data suggest that, in addition to commonly used conventional assay methods (such as ALP activity or alizarin red S staining), live cell imaging of osteoblasts on a surface and analysis of cell movement could serve as effective evaluation criteria for compatibility of bone with an implant surface.

## Conclusions

Once osteoblasts sense a need for bone formation, they proliferate and migrate to a site optimized for such activity. Our data suggest that a combination of osteogenic molecules (such as DSG4 and PRDX6) and surface geometry (such as polygonal septa of calcified cartilage) may define sites optimal for initiation of bone formation during endochondral ossification.

## Supporting Information Available

List of primers and the Universal Probe Library (UPL) used in this study (Table S1); screening of osteogenic factors by mineralization assay; Alizarin red S staining (Figure S1); effect of treatment with recombinant DSG4 and PRDX6 proteins on osteoblast differentiation (Figure S2); amino acid conservation of DSG4 and PRDX6 proteins (Figure S3); and three different arrangements of micropatterns used for mineralization assays (Figure S4) (PDF)
