# Supplementary material for: Calcified Cartilage-Guided Identification of Osteogenic Molecules and Geometries
Source: ACS Biomater Sci Eng. 2024 Apr 18;10(5):2983–94. doi: 10.1021/acsbiomaterials.3c01799 (PMC11094677; doi:10.1021/acsbiomaterials.3c01799)

Supplementary Table and Figures for

Calcified cartilage-guided identification of osteogenic molecules and geometries

Katsuhiro Kawaai[1], Yukiko Kuroda[1], and Koichi Matsuo[1]

[1]Laboratory of Cell and Tissue Biology, Keio University School of Medicine, Tokyo, Japan.

*Corresponding author. Email: k-kawaai@keio.jp

Number of Pages: 6(Pages S1-S6)
Number of Tables: 1 (Table S1)
Number of Figures: 4 (Figure S1-S4)

Table S1. List of primers and Universal Probe Library (UPL) used in this study

Gene	Primer name	5'-Sequence-3'	Purpose	
Lrrc15	BamHI-Lrrc15-ATG-S	tcggatcc**atg**ccgctgaaacattatctcc	for cloning	full-length
Lrrc15	NotI-Lrrc15-AS	gagcggccgccgcactcattgggagccttcatctg	for cloning	full-length
Lrrc15	BamHI-Lrrc15(delSP)-ATG-S	atggatcctact**atg**gctgccccagcgaatg	for subcloning	Extracellular domain without SP
Lrrc15	NotI-Lrrc15(delTM)-AS	gagcggccgcccccactctgggcatcagtc	for subcloning	Extracellular domain without SP
Dsg4	BamHI-Dsg4(delSP)-S	atggatccgagtggatcaagtttgcggcag	for subcloning	Extracellular domain without SP
Dsg4	NotI-Dsg4(delTM)-AS	gagcggccgccgccaagtccgacgtttgactg	for subcloning	Extracellular domain without SP
Dsg4	SalI-pmeI-mycHis-AS	ctgtcgacgtttaaactcaatggtgatggtg	for subcloning	Extracellular domain without SP
Prdx6	EcoRI-Prdx6-ATG-S	cggaattc**atg**cccggagggttgcttctc	for cloning	full-length
Prdx6	SalI-Prdx6-AS	gagtcgac**tta**aggctggggtgtataacg	for cloning	full-length
Sprr1b	EcoRI-Sprr1b-ATG-S	cggaattc**atg**agttcacatcagcagaagcag	for cloning	full-length
Sprr1b	SalI-Sprr1b-AS	gagtcgac**cta**cttttgctttgtcttctgttg	for cloning	full-length
Gapdh	UPL Mouse GAPD Gene assay	Roche cat. no. 05046211001	qPCR	UPL + Primers Kit
Col1a1	UPL #15	Roche cat. no. 04685148001	qPCR	
Col1a1	mCol1a1_UPL_left	catgttcagctttgtggacct	qPCR	amplicon: 94
Col1a1	mCol1a1_UPL_right	gcagctgacttcagggatgt	qPCR	
Alp1	UPL #12	Roche cat. no. 04685113001	qPCR	
Alp1	mAlpl_UPL_left	cggatcctgaccaaaaacc	qPCR	amplicon: 74
Alp1	mAlpl_UPL_right	tcatgatgtccgtggtcaat	qPCR	
Bglap	UPL #32	Roche cat. no. 04687655001	qPCR	
Bglap	mBglap_UPL_left	agactccggcgctacctt	qPCR	amplicon: 93
Bglap	mBglap_UPL_right	ctcgtcacaagcagggttaag	qPCR	

underlining, restriction site; bold, start or stop codon   SP, Signal peptide

Figure S1

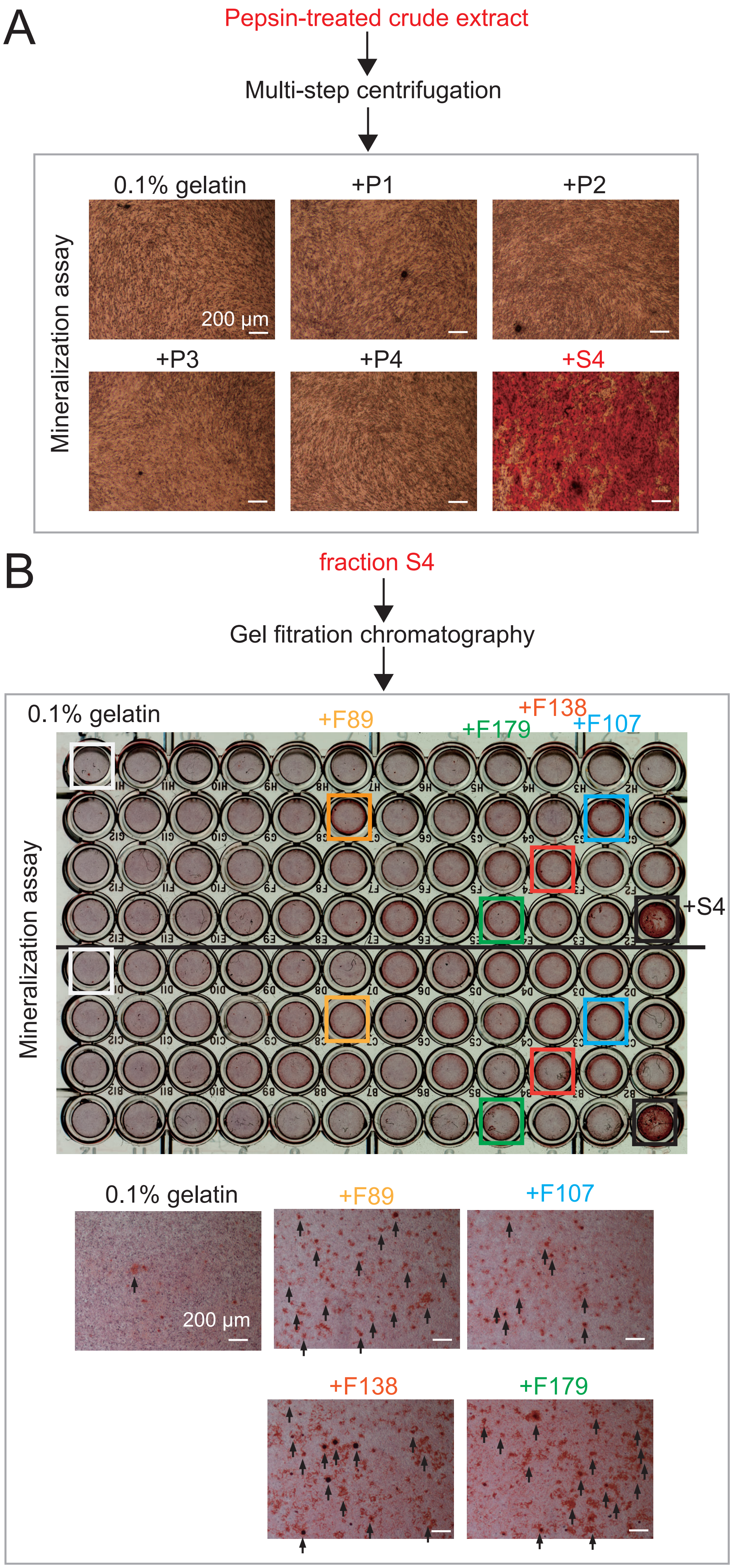
A
Pepsin-treated crude extract
Multi-step centrifugation
Mineralization assay
0.1% gelatin
+P1
+P2
+P3
+P4
+S4
200 μm
B
fraction S4
Gel fitration chromatography
0.1% gelatin
+F89
+F179
+F138
+F107
+S4
Mineralization assay
0.1% gelatin
+F89
+F107
+F138
+F179
200 μm

Figure S2

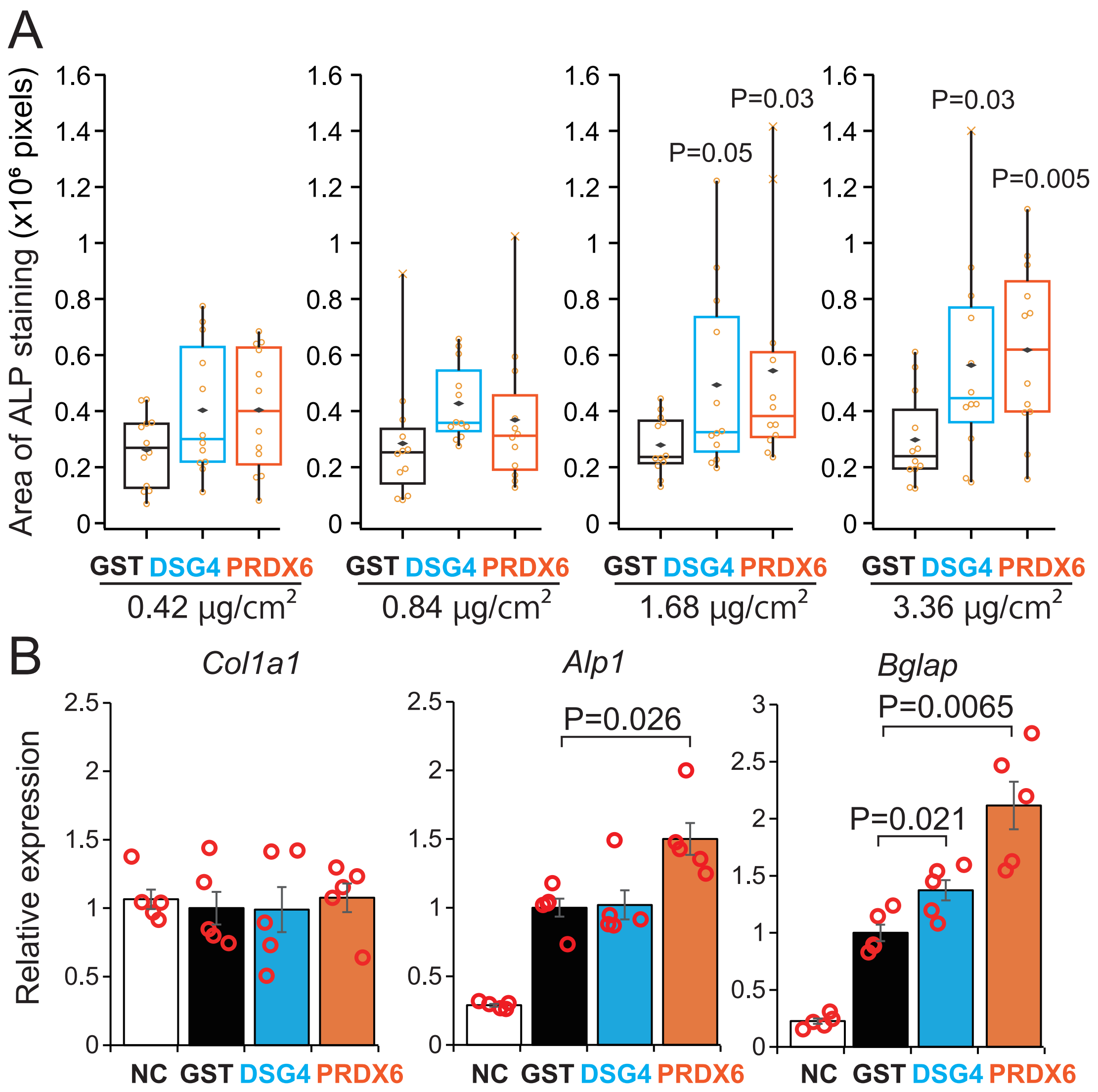

A
Area of ALP staining (x10⁶ pixels)
1.6
1.4
1.2
1
0.8
0.6
0.4
0.2
0
P=0.05
P=0.03
P=0.03
P=0.005
GST DSG4 PRDX6
0.42 μg/cm2
0.84 μg/cm2
1.68 μg/cm2
3.36 μg/cm2
B
Col1a1
Alp1
Bglap
Relative expression
2.5
2
1.5
1
0.5
0
3
P=0.026
P=0.0065
P=0.021
NC GST DSG4 PRDX6

Figure S3

A

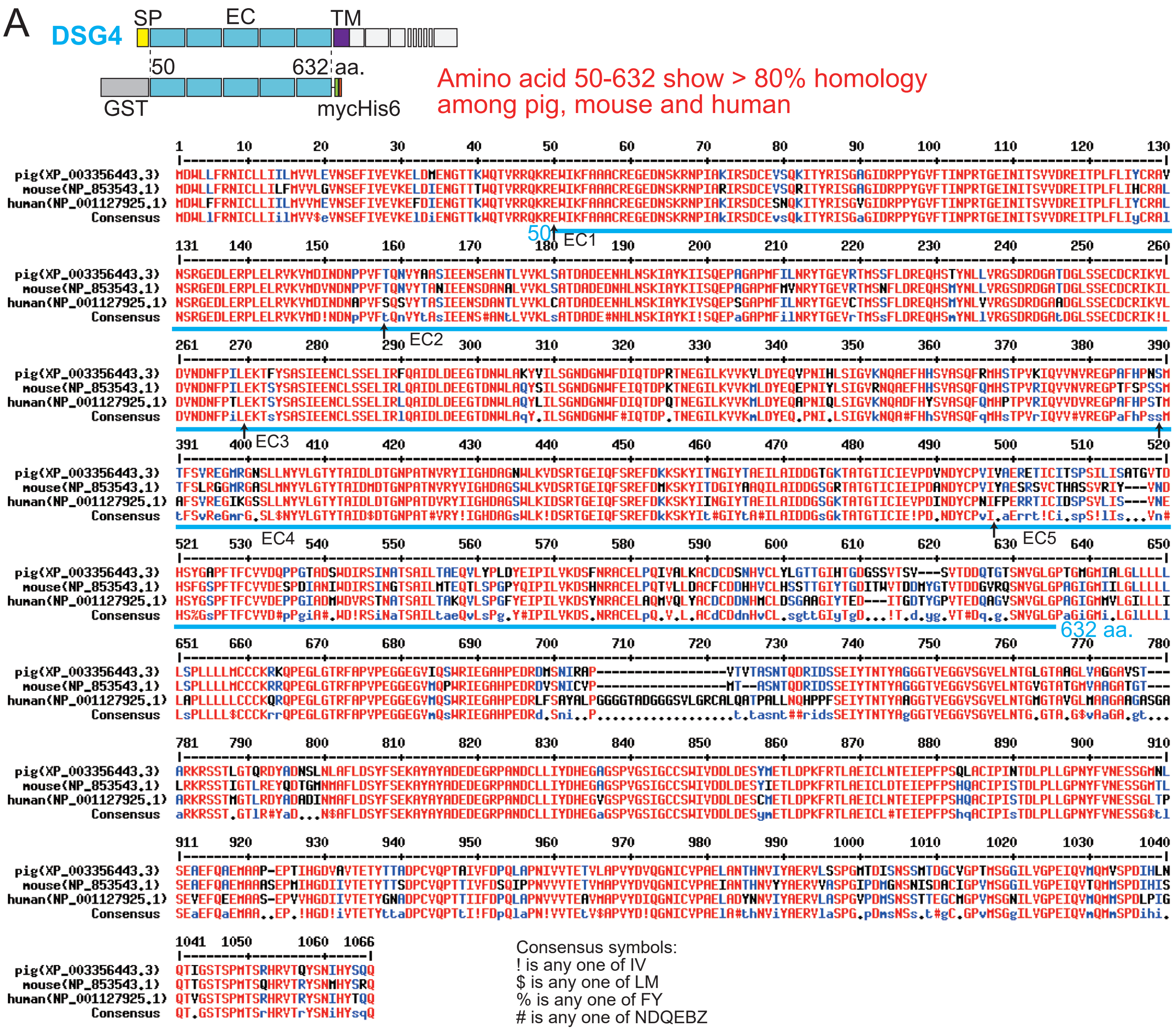
DSG4
SP
EC
TM
50
632 aa.
GST
mycHis6
Amino acid 50-632 show > 80% homology among pig, mouse and human
pig(XP_003356443.3)
mouse(NP_853543.1)
human(NP_001127925.1)
Consensus
EC1
EC2
EC3
EC4
EC5
Consensus symbols:
! is any one of IV
$ is any one of LM
% is any one of FY
# is any one of NDQEBZ

B

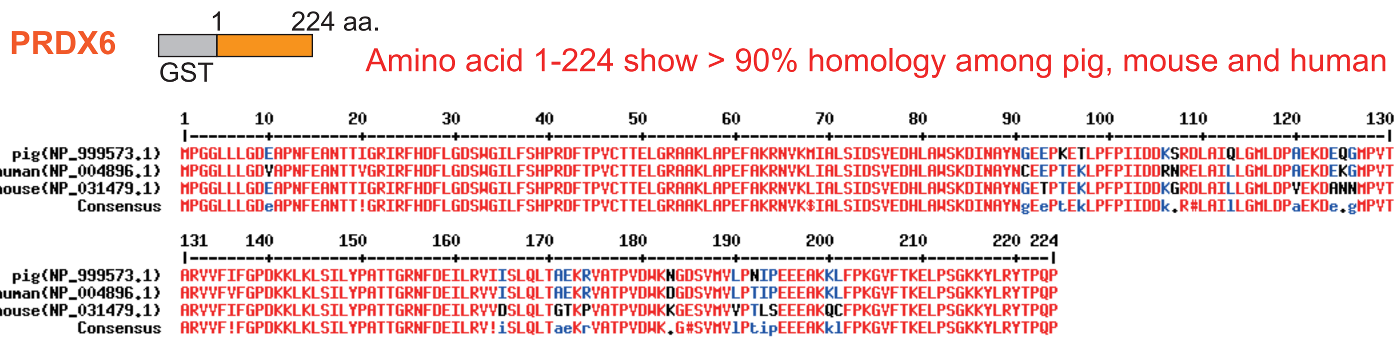
PRDX6
1
224 aa.
GST
Amino acid 1-224 show > 90% homology among pig, mouse and human
pig(NP_999573.1)
human(NP_004896.1)
mouse(NP_031479.1)
Consensus

Figure S4

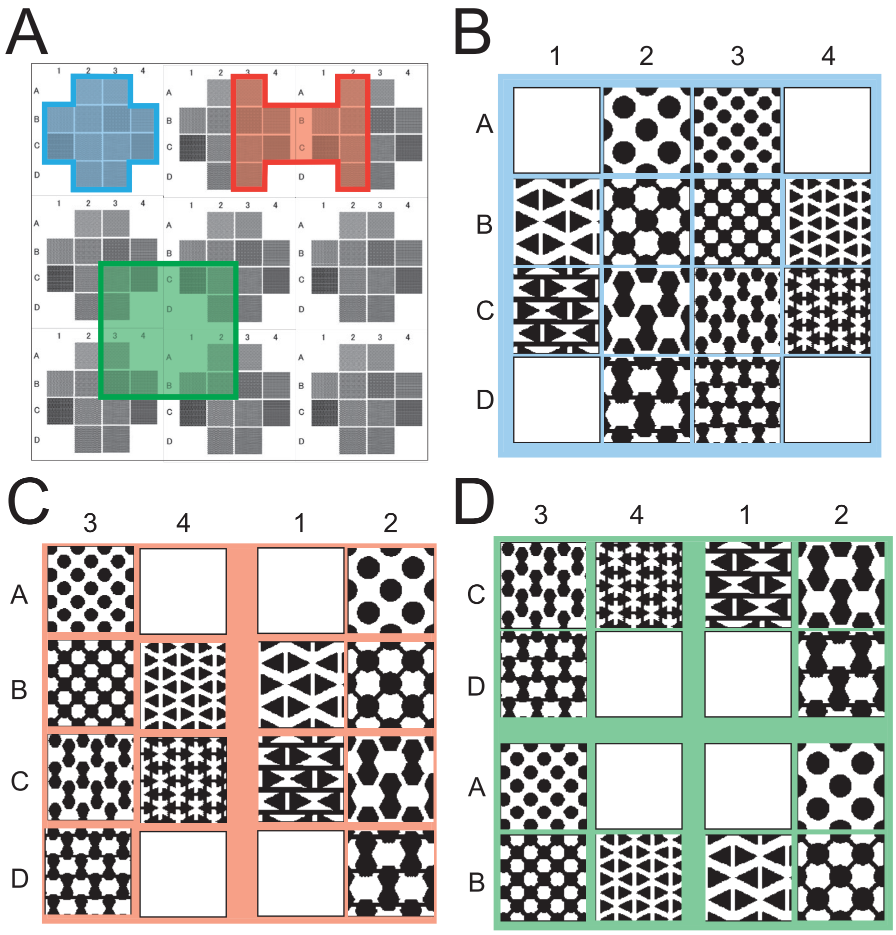

Supplement: Supplementary file 1 — ab3c01799_si_001.pdf [file ab3c01799_si_001.pdf]
